# Two Novel Functional Mutations in Promoter Region of *SCN3B* Gene Associated with Atrial Fibrillation

**DOI:** 10.3390/life12111794

**Published:** 2022-11-05

**Authors:** Liyan Lin, Ke Li, Beijia Tian, Mengru Jia, Qianyan Wang, Chengqi Xu, Liang Xiong, Qing Wang, Yali Zeng, Pengyun Wang

**Affiliations:** 1 Department of Clinical Laboratory, Liyuan Hospital, Tongji Medical College, Huazhong University of Science and Technology, Wuhan 430077, China; 2 Key Laboratory of Molecular Biophysics of the Ministry of Education, Cardio-X Institute, College of Life Science and Technology, Huazhong University of Science and Technology, Wuhan 430074, China; 3 Human Genome Research Center, College of Life and Science and Technology, Huazhong University of Science and Technology, Wuhan 430074, China; 4 Liyuan Cardiovascular Center, Liyuan Hospital, Tongji Medical College, Huazhong University of Science and Technology, Wuhan 430077, China

**Keywords:** atrial fibrillation, mutation, *SCN3B*, GATA4

## Abstract

The sodium voltage-gated channel beta subunit 3 (*SCN3B*) plays a crucial role in electrically excitable cells and conduction tissue in the heart. Some previous studies have established that genetic modification in sodium voltage-channel genes encoding for the cardiac β-subunits, such as *SCN1B*, *SCN2B*, *SCN3B* and *SCN4B*, can result in atrial fibrillation (AF). In the current study, we identified two rare variants in 5′UTR (NM_018400.4: c.-324C>A, rs976125894 and NM_018400.4: c.-303C>T, rs1284768362) of *SCN3B* in two unrelated lone AF patients. Our further functional studies discovered that one of them, the A allele of c.-324C>A (rs976125894), can improve transcriptional activity and may raise *SCN3B* expression levels. The A allele of c.-324C>A (rs976125894) has higher transcriptional activity when it interacts with GATA4, as we confirmed transcription factor GATA4 is a regulator of *SCN3B*. To the best of our knowledge, the current study is the first to demonstrate that the gain-of-function mutation of *SCN3B* can produce AF and the first to link a mutation occurring in the non-coding 5′UTR region of *SCN3B* to lone AF. The work also offers empirical proof that GATA4 is a critical regulator of *SCN3B* gene regulation. Our findings may serve as an encyclopedia for AF susceptibility variants and can also provide insight into the investigation of the functional mechanisms behind AF variants discovered by genetic methods.

## 1. Introduction

Atrial Fibrillation (AF), characterized by a rapid and chaotic rhythm in atria, including the absence of P waves, irregular RR intervals and a fast-atrial rate of up to 300 beats per minute, is the most common clinically sustained cardiac arrhythmia [1]. AF occurs when the normal cycle of electrical impulses is interrupted, resulting in a disorganized quiver of the atria and an irregular contraction of the ventricles. With a 0.7% population prevalence, AF affects about 8 million people in China, 2.2 million people in the United States and 4.5 million people in the European Union. The estimated frequency of AF in people over 65 is 8.6% and its incidence has remained much higher in the elderly population [2]. The risk of stroke is increased five-fold by AF, while the risk of congestive heart failure is increased three-fold. AF is also a well-known independent risk factor for stroke and can aggravate heart failure. According to recent data, AF doubles to triples the chance of sudden cardiac death [3].

AF is often associated with complications, such as coronary artery disease (CAD), hypertension, valvular heart disease, hyperthyroidism, heart failure and structural heart diseases. In the Framingham cohort and after age adjustment, hypertension, diabetes mellitus, coronary artery disease and smoking were the main risk factors for non-valvular AF [4]. The combined effects of these factors could explain 44% of the burden of non-valvular AF in men and 58% in women. In some of the cases, AF occurs in the absence of the complications of hypertension, heart failure, coronary heart diseases or other types of cardiac diseases, and is, hence, clinically diagnosed as lone AF [3].

Epidemiological studies revealed that while numbers of prevalent cardiovascular disorders are risk factors for developing AF, genetic components have also been proven to be involved, particularly in the case of lone AF [5]. According to a study of Danish twins, the heritability of AF was as high as 62% [6]. The discovery of a few private, AF-causing mutations in ion channel genes highlights the significant contribution of genetic factors to the etiology of AF. The S140G mutation in the ion channel gene *KCNQ1* was first reported by Chen et al. in 2003 [7]. Later, mutations in non-ion channel genes, such as *NPPA* and *NUP155*, were found in some patients with lone AF [8,9]. Mutations or rare variants in more than 40 genes are believed to be the cause of familial or sporadic AF and these genes were mapped to pathways of ion channels, ion channel-associated genes, transcription factors, cardiac structural components, signaling regulators and others [10,11]. Loss-of-function rare variants in the *TTN* gene are enriched among individuals with early-onset AF (2.1% prevalence) versus control subjects (1.1% prevalence; odds ratio, 1.76 (95% CI, 1.04–2.97)) [12]. In addition to the mutations and rare variants, genome-wide association studies (GWAS) have been performed for AF in several populations, which successfully identified about 300 common variants associated with AF [11].

Studies have linked cardiac arrhythmogenesis to mutations in the genes encoding the cardiac sodium channel (Na_V_1.5) and AF-causing variants have been reported in both the α-subunit (encoded by the *SCN5A* gene) and associated β-subunits (Navβ) (encoded by the genes *SCN1B–SCN4B*) [13,14,15,16,17]. *SCN1B*, the gene encoding for the β1-subunit and *SCN2B,* the gene encoding for the β2-subunit, have been identified as susceptibility genes for AF [14]. *SCN1B* and *SCN3B* that encode for the β3-subunit have been identified as susceptibility genes of Brugada Syndrome (BrS), while *SCN4B* has been linked to the congenital long-QT syndrome (LQTS) 3, ventricular tachycardia and familial AF [15,16,17,18]. In our previous study, A103V mutation in *SCN3B* was found to induce lone AF and Olesen et al. also showed that R6K, L10P and M161T mutation of *SCN3B* was associated with early-onset lone AF [19,20]; however, there are no more reports that link *SCN3B* mutation to AF.

In the current study, we carried out mutation screening of the *SCN3B* gene in 355 lone AF patients and found that two patients carried rare variants in 5′UTR (NM_018400.4: c.-324C>A, rs976125894 and NM_018400.4: c.-303C>T, rs1284768362) of *SCN3B* and our further functional studies revealed that the rare variant c.-324C>A (rs976125894), which is located in the promoter region and enhanced the transcriptional activity, may regulate the expression level of *SCN3B* through interacting with the GATA4 transcription factor.

## 2. Materials and Methods

### 2.1. Study Population

The study subjects included in the present study were enrolled from the GeneID database. All the study patients are of Han ethnic origin by self-description.

AF diagnosis was performed by a panel of cardiologists based on the data from electrocardiograms (ECG) and/or Holter ECG and based on the criteria of the ACC/AHA/ESC AF guidelines. The ECG characteristics of AF include the absence of P waves, replacement of P waves by rapid oscillations of fibrillation waves that varied in size, shape and timing (referred to as “f” waves) and irregular ventricular response (irregular RR intervals) with abnormal atrioventricular conduction. Patients with other types of cardiac arrhythmias (e.g., ventricular arrhythmias), thyroid dysfunction, congenital heart disease, cardiomyopathies and valvulopathies were excluded. An AF patient was diagnosed as being affected with “lone AF” if he/she had no CAD, congestive heart failure, hypertension or diabetes and was below the age of 55 years at the first diagnostics of AF.

Demographic and clinical information on age, gender, CAD, hypertension and diabetes mellitus was obtained from medical records. CAD was defined as ongoing therapy of CAD, 70% luminal narrowing in at least one vessel by coronary angiography, percutaneous coronary angioplasty, coronary artery bypass graft and MI. Hypertension was defined as ongoing medication for hypertension, systolic blood pressure of ≥140 mmHg or diastolic blood pressure of ≥90 mmHg. Diabetes mellitus was defined as ongoing therapy of diabetes or a fasting plasma glucose level of ≥7.0 mmol/L.

The study protocol was approved by Ethics Committee on Human Subject Research at Huazhong University of Science and Technology (HUST) and Liyuan Hospital Affiliated to Tongji Medical College of HUST and also conforms to the principles outlined in the Declaration of Helsinki. Written informed consent was obtained from all subjects.

### 2.2. Mutation Analysis of SCN3B

Genomic DNA was isolated from peripheral blood samples using standard protocols with the Wizard Genomic DNA Purification Kit (Promega, Madison, WI, USA). Direct Sanger DNA sequencing was used for screening mutations as described previously. All the six exons of *SCN3B* (NM_018400), including the coding region, 5′ untranslated region, 3′ untranslated region and the boundaries of intron–exon were amplified by polymerase chain reactions (PCR) from AF patient DNA samples and sequenced. The primers for PCR are shown in Table 1. Mutation analysis of the *SCN3B* gene was performed in all study subjects to identify a novel mutation responsible for AF. A variant was defined as a mutation if present in less than 0.01% in the general population according to the public database.

The sequencing of *SCN3B* in unrelated controls was checked by high-resolution melt analysis (HRM) utilizing a Rotor-Gene 6000 High-Resolution Melt system (Corbett Life Science, Sydney, NSW, Australia), as previously disclosed by us, to validate the mutation frequency in the Chinese common population. Primers for PCR of HRM are shown in Table 1. Ten samples were randomly selected for direct Sanger sequencing to verify the accuracy of HRM genotyping.

### 2.3. Bioinformatics-Based Prediction

The deleterious potential of the identified mutations was measured using Functional Analysis through Hidden Markov Models (FATHMM) (http://fathmm.biocompute.org.uk/ accessed on 5 August 2022), GERP++ (http://mendel.stanford.edu/SidowLab/downloads/gerp/index.html accessed on 5 August 2022) and regulatory Mendelian mutation (ReMM) (https://charite.github.io/software-remm-score.html accessed on 5 August 2022).

The possible regulatory features of the variants in the non-coding region were predicted by Ensembl database (http://asia.ensembl.org/ accessed on 5 August 2022) and ENCODE data of UCSC genome browser (https://genome.ucsc.edu accessed on 5 August 2022). The Human Transcription Factor Database (http://bioinfo.life.hust.edu.cn/HumanTFDB#!// accessed on 15 August 2022) and JASPAR (https://jaspar.genereg.net accessed on 15 August 2022) were applied to analyze and predict the potential transcription factors binding to the area of the SNP.

### 2.4. Clones and Site-Directed Mutagenesis

To further explore whether the genomic region overlapping > (rs976125894) or c.-303C>T (rs1284768362) has regulatory activity and is a potential promoter, the wild-type 858 bp length genomic fragment overlapping rs976125894 and rs1284768362 was generated by PCR analysis using human genomic DNA as a template. The Prime STAR HS DNA polymerase (Takara, Dalian, China) was applied and PCR primers were SCN3B-Kpn I-F 5′-GATTCCCAGGGCTGACAGCACACACGG-3′ and SCN3B-HindⅢ-R 5′-AAGCTTGAAGCCGCCAGCCCCAGAAG-3′. The wild-type 858 bp length PCR products were digested with restriction enzyme KpnI and HindIII (Takara, Dalian, China) and then sub-cloned into the multiple cloning site of the pGL3-basic luciferase vector (Promega, Madison, WI, USA), resulting in pGL3-SCN3B-WT.

The two variants, c.-324 C>A (rs976125894) and c.-303 C>T (rs1284768362), were introduced into the pGL3-SCN3B-WT plasmid by using PCR-based site-directed mutagenesis, referred to as pGL3-Mut-324A and pGL3-Mut-303T, respectively. The primer sequences for c.-324 C>A were 5′-GAGCGAGGCAGGGGAGCGAGTGGAAGCTGG-3′ (forward) and 5′-CCAGCTTCCACTCGCTCCCCTGCCTCGCTC (reverse). The primer sequences for c.-303 C>T were 5′-AGTGGAAGCTGGAGTTCTGGGGTGGGCGGGGAGGC-3′ (forward) and 5′-GCCTCCCCGCCCACCCCAGAACTCCAGCTTCCACT-3′ (reverse). The PCR products were digested by DpnⅠ (Takara, Dalian, China) in a 37 °C incubator for 6 h.

The 5′ UTR contained wild-type c.-324C or mutant A, plus the whole CDS (delete the stop codon) of SCN3B was amplified and digested by EcoRI and BamHI (Takara, Dalian, China) and then sub-cloned into pEGFP-N1 vector (named pEGFP-N1-WT-324C and pEGFP-N1-Mut-324A) before the ORF of EGFP to validate whether the mutant can regulate the expression of SCN3B. The primer sequences are 5′-GGCGACCGGTGGATCCCGTTCCTCCACTGGTACCGCAGAG-3′ (forward) and 5′-CTCAAGCTTCGAATTCTGCAGGGCTGACAGCACAC-3′ (reverse).

pcDNA3.1, pcDNA3.1-GATA4, pcDNA3.1-IKZF1 and pcDNA3.1-WT1 were reserved in the plasmid library from our laboratory. All constructions were confirmed by Sanger sequencing.

### 2.5. Dual Luciferase Reporter Assays

Human embryonic kidney 293T cells (HEK293T) and rat cardiomyoblast cells (H9C2) were cultured in 24-well plates, at a density of 10^5^ cells per well and transfected at 37 °C with 5% CO_2_ of 24 h seeding in Dulbecco’s modified Eagle’s medium (DMEM, Gibco, USA) supplemented with 10% fetal bovine serum (FBS, Gibco, USA). Further, 25ng Renilla luciferase control vector was co-transfected with (1) 200ng pGL3-basic, (2) 200ng pGL3-WT, (3) 200ng pGL3-Mut-324A or 200ng pGL3-Mut-303T. For transcription factor co-transfection experiments, 25ng Renilla luciferase control vector was co-transfected with (1) 100ng pcDNA3.1 + 100ng pcDNA3.1-GATA4 or pcDNA3.1-IKZF1 or pcDNA3.1-WT1, (2) 100ng pGL3-WT + 100ng pcDNA3.1-GATA4 or pcDNA3.1-IKZF1 or pcDNA3.1-WT1, (3) 100ng pGL3-Mut-324A + 100ng pcDNA3.1-GATA4 or pcDNA3.1-IKZF1 or pcDNA3.1-WT1. Lipofectamine 2000 (Invitrogen, Thermo Fisher Scientific, Waltham, MA, USA) was used. Cells were harvested in 48 h. Firefly luciferase signal intensities and Renilla luciferase activities were measured by using the Dual-Glo Luciferase Assay System (Promega, Madison, WI, USA) on GloMax 20/20 (Promega, Madison, WI, USA). Renilla luciferase activity was normalized by relative luciferase intensity. Independent experiments were performed in triplicate at minimum for wild types and mutant types.

### 2.6. Western Blotting

HEK293T cells were plated in 12-well plates in a humidified incubator at 37 °C and 5% CO_2_. After 24 h seeding in DMEM with 10% of FBS, at a density of 10^5^ cells per well, cells were transfected with (1) 1 μg pEGFP-N1, (2) 1 μg pEGFP-N1-WT-324C, (3) 1 μg pEGFP-N1-Mut-324A, (4) 500 ng pEGFP-N1 + 500 ng pcDNA3.1-GATA4, (5) 500 ng pEGFP-N1-WT-324-C + 500 ng pcDNA3.1-GATA4 and (6) 500 ng pEGFP-N1-Mut-324A + 500 ng pcDNA3.1-GATA4. Lipofectamine 2000 was used. Cells were collected using cell lysis buffer after 48 h feeding. SCN3B (Novus, Littleton, CO, USA) and GAPDH (Abcam, Cambridge, UK) antibodies were used for Western blotting.

### 2.7. RNA Isolation and Quantitative RT-PCR

Total RNA was isolated with RNAiso Plus TRIZOL (Takara, Dalian, China) and was reverse transcribed using HiScriptⅢ RT SuperMix for qPCR (Vazyme, Nanjing, China), according to the manufacturer’s protocol. qRT-PCR analysis was conducted using MonAmp qPCR SYBR Green Mix (Monad, Suzhou, China) in an Applied Biosystems instrument (Thermo Fisher, Waltham, MA, USA). qPCR-primers are listed in Table 2.

### 2.8. Statistical Analysis

For analysis of promoter activity, data were presented as means ± standard deviations from at least three independent experiments. Statistical multiple-group comparisons were evaluated with a one-way ANOVA test with Turkey’s post hoc test and the difference between two groups was tested using Student’s unpaired *t*-test. A value of *p* < 0.05 was considered statistically significant. Data analysis was performed using GraphPad Prism software version 6 (GraphPad 6, San Diego, CA, USA).

## 3. Results

### 3.1. Identification of Two Novel Susceptibility Mutations in SCN3B

We performed a mutation screening of *SCN3B* in 355 lone AF sporadic patients selected from the GeneID database to confirm whether *SCN3B* is the causative gene for AF. The basic clinical characteristics of the study patients are shown in Table 3.

**Figure 1:**
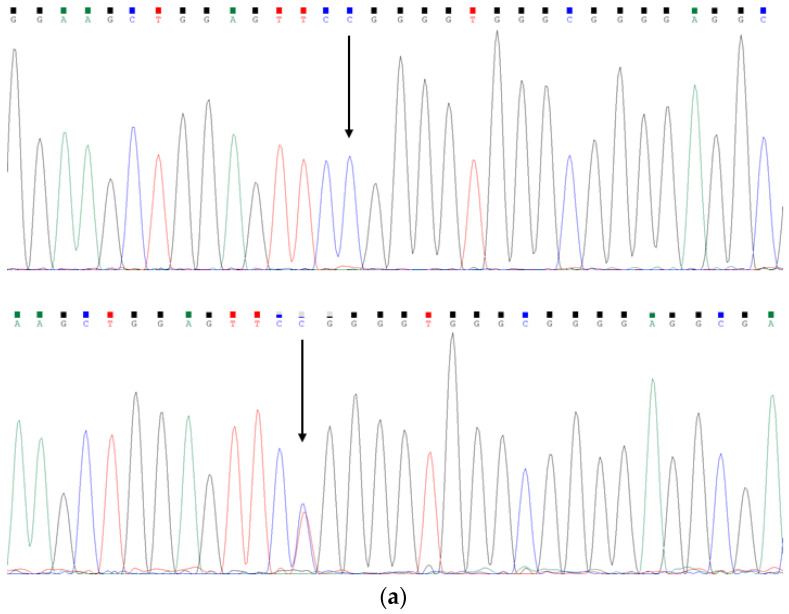

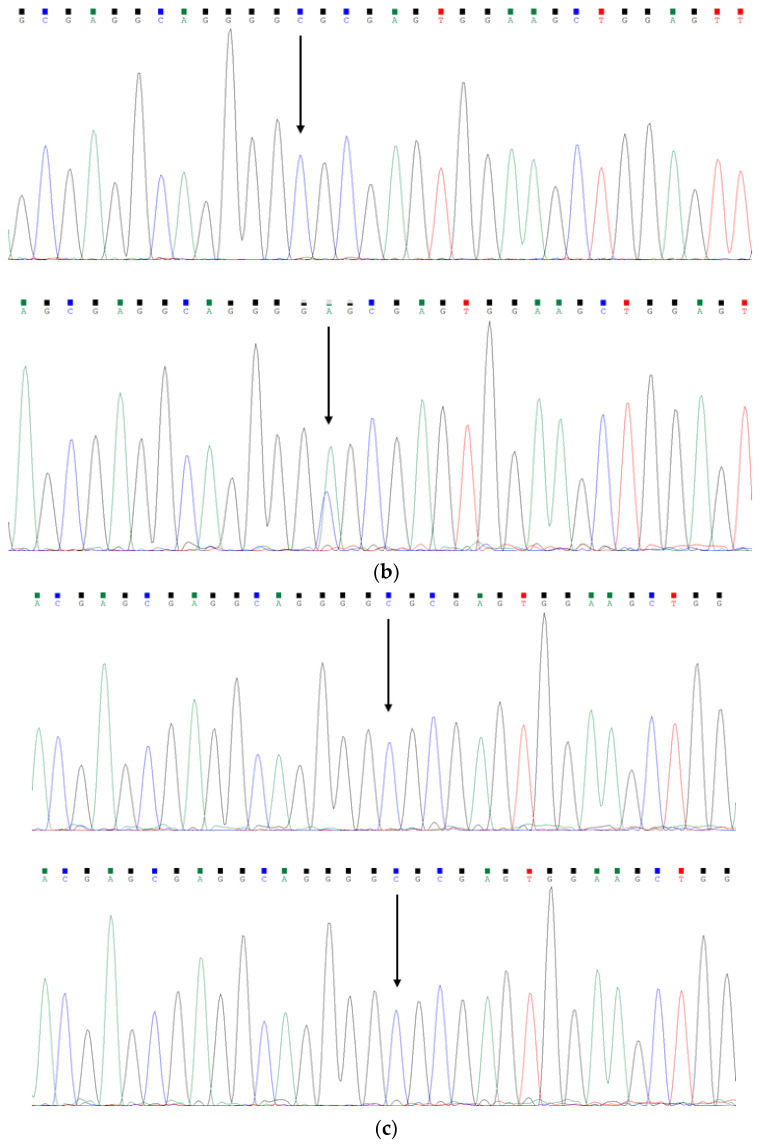
The sequence electropherograms of *SCN3B* in two lone AF patients and the parents of subject who carried c.-324>A (rs976125894) mutation. The arrow indicates the heterozygous *SCN3B* mutations of (**a**) c.-303C>T (rs1284768362, down) and (**b**) c.-324C>A (rs976125894, down), compared with control sequences (up), respectively; (**c**) the results of Sanger sequencing of the parents of subject with c.-324>A (rs976125894) mutation (mother, up; father, down).

**Figure 2:**
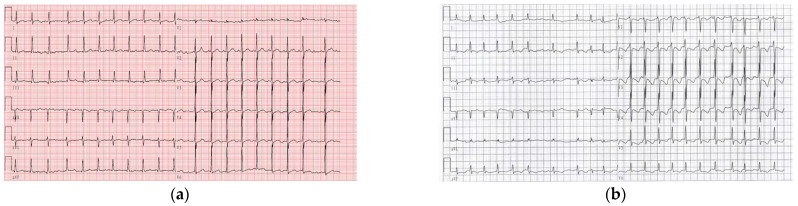
The ECGs of the two lone AF patients. (**a**) ECG of the patient who carried c.-303C>T (rs1284768362); (**b**) ECG of the patient who carried c.-324C>A (rs976125894).

After filtering by the allele frequency (<0.01% in the general population according to the public database), we identified two susceptibility rare variants, c.-324C>A (NM_018400.4, rs976125894) and c.-303C>T (NM_018400.4, rs1284768362), in the first exon and the 5′UTR region of *SCN3B* gene in two unrelated patients clinically diagnosed as lone AF (Figure 1a,b). The patient who carried the c.-324C>A mutation (rs976125894) is a female who was first discovered to have lone AF at the age of 41 and the patient who carried the c.-303C>T mutation (rs1284768362) is a man who was first found to have lone AF at the age of 46. We tried to obtain genomic DNA samples from each parent of these two *SCN3B* mutation carriers in order to determine if the mutations were *de novo* or germline inherited. While DNA from the parents of subjects who carried s c.-303C>T mutation (rs1284768362) was not accessible, we were successful in getting genomic DNA from the parents of the subject who carried the c.-324C>A (rs976125894) mutation. The results demonstrated that the mutation c.-324C>A (rs976125894) originated *de novo* as the parents showed an homozygous wild-type genotype and without AF (Figure 1c). The ECGs of two patients are shown in Figure 2.

According to the public database, both variants were found in exon 1 and were incredibly rare in the general population. In the public databases, such as the gnomAD database (1 allele in mutation carriers in a total of 152,180 alleles, with a frequency of 6.57 × 10^−6^) and the TOPMED database (1/125568, 7.96 × 10^−6^), the frequency of the mutation A allele of the c.-324C>A (rs976125894) variant was incredibly low. For the c.-303C>T (rs1284768362) variant, there was no record in the gnomAD database and we found only two carriers in the TOPMED database (2/125568). We also performed validation of the frequency of the two variants in the Chinese Han population and the results indicated that none of the mutations were present in control group containing 500 subjects without AF.

Using software that can predict the effects of non-coding variants, we conducted in silico analyses to determine whether the mutations are deleterious. The results indicated that the mutation c.-324C>A (rs976125894) may be deleterious based on the fathmm MKL non-coding score (0.993), GERP++ RS rankscore (0.997) and ReMM score of 0.984. Only fathmm MKL score revealed deleterious effects (0.915) for the c.-303C>T (rs1284768362) variant, but not in others.

### 3.2. Transcriptional Activity and Expression Level Enhanced in the Alternative a Allele of Rare Variant c.-324C>A (rs976125894)

**Figure 3:**
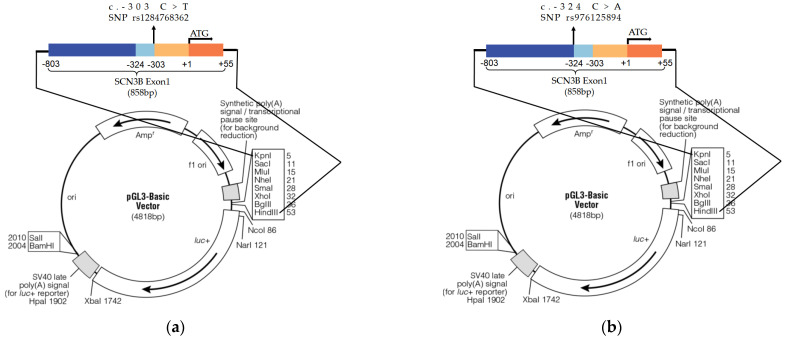
The 858 bp exon1 of *SCN3B* containing (**a**) rs1284768362 C/T and (**b**) rs976125894 C/A was directionally cloned into pGL3-basic vector between the KpnI and HindIII sites.

Both the identified AF-related variants, rs1284768362 and rs976125894, are in the 5′UTR of *SCN3B* mRNA. The regulatory feature records in the Ensembl database indicated that both rs1284768362 and rs976125894 were active promoters in several tissues and cell types, including cardiac muscle (Ensembl regulatory feature ENSR00000445310). We next assess whether the mutant alleles of these two 5′-UTR rare variants alter the *SCN3B* promoter activity by in vitro cell transfection studies using luciferase reporter assays in HEK293T cells and H9C2 cells. We cloned the whole exon 1 of *SCN3B* (858 bp) into the pGL3-basic plasmid and then it was used as a template to construct a c.-303 C>T (rs1284768362) plasmid (Figure 3a) and a c.-324 C>A (rs976125894) plasmid (Figure 3b) by PCR-based site-directed mutagenesis.

**Figure 4:**
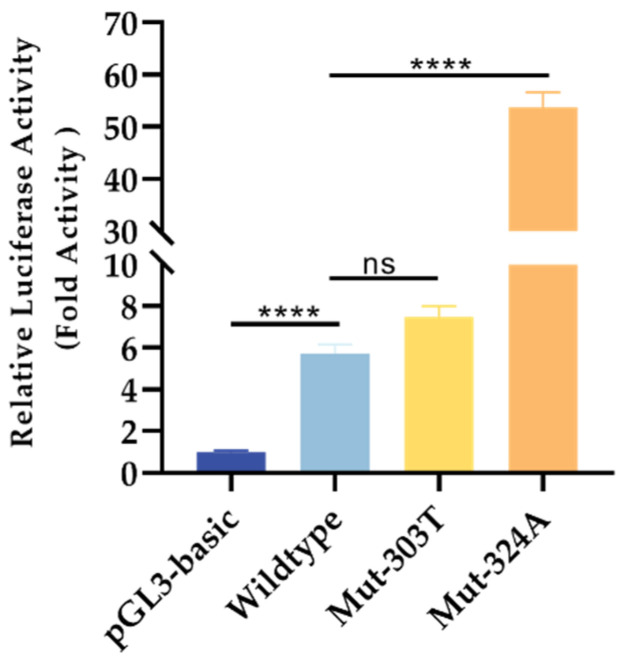
Dual luciferase assay manifests that the wild-type construction has promoter activity and rs976125894 with A allele has a significantly higher transcriptional activity when compared to the wild-type one. A one-way ANOVA test with Turkey’s post hoc test was used to compare difference among groups. **** *p* < 0.0001, ns, not significant.

Dual luciferase reporter assays were applied to validate the functionality of the two variants. Results of the dual luciferase reporter assays confirmed that c.-324C>A variant (rs976125894), carrying a reference C allele, has promoter activity relative to the pGL3-basic vector. As to the mutant A allele carriers, the relative luciferase intensity is significantly enhanced, compared to that of the wild-type C carriers. For the c.-303 C>T (rs1284768362) variant, there is no significant discrepancy between the reference C allele carriers and the alternative T allele carriers (Figure 4).

**Figure 5:**
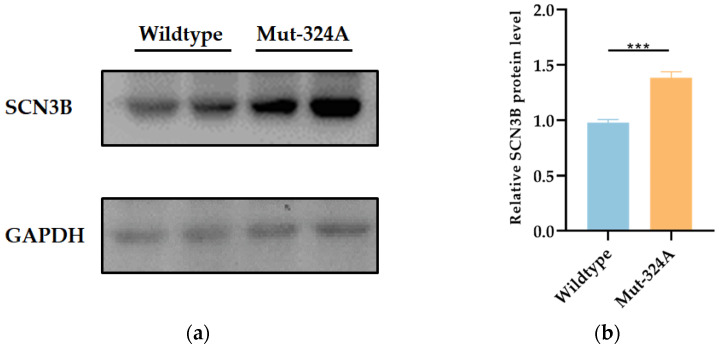
(**a**) Immunoblot shows the expression level of SCN3B between the wild type and the mutant type; (**b**) qualification of SCN3B expression level from (**a**). Three repeats of each experiment were conducted and analyzed by Student’s unpaired *t*-test. *** *p* < 0.001.

Further Western blot analysis was then performed to evaluate whether the c.-324C>A (rs976125894) regulates the expression level of SCN3B in HEK293T cells. Compared with the wild-type allele C, the promoter region containing the mutant A allele is able to promote higher protein expression level of SCN3B (Figure 5), which is in line with the results of luciferase reporter assays we obtained. These findings imply that the mutation at position-324 from C to A has an elevated effect on the transcriptional activity of the *SCN3B* promoter, suggesting that the variant c.-324C>A variant (rs976125894) is further suspected as disease inducing.

### 3.3. c.-324C>A Variant (rs976125894) Regulates the Expression of SCN3B through Interacting with Transcription Factor GATA4

**Figure 6:**
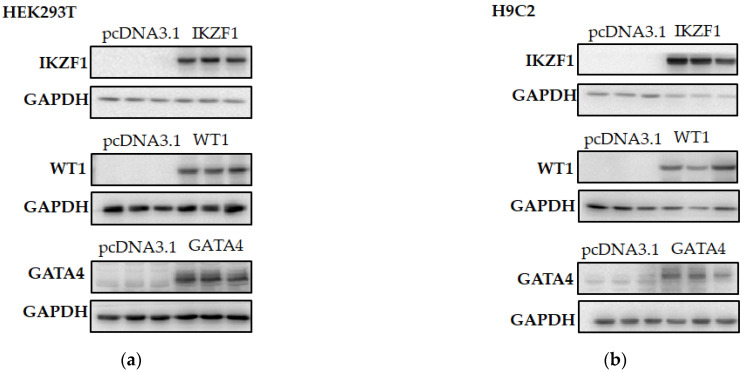

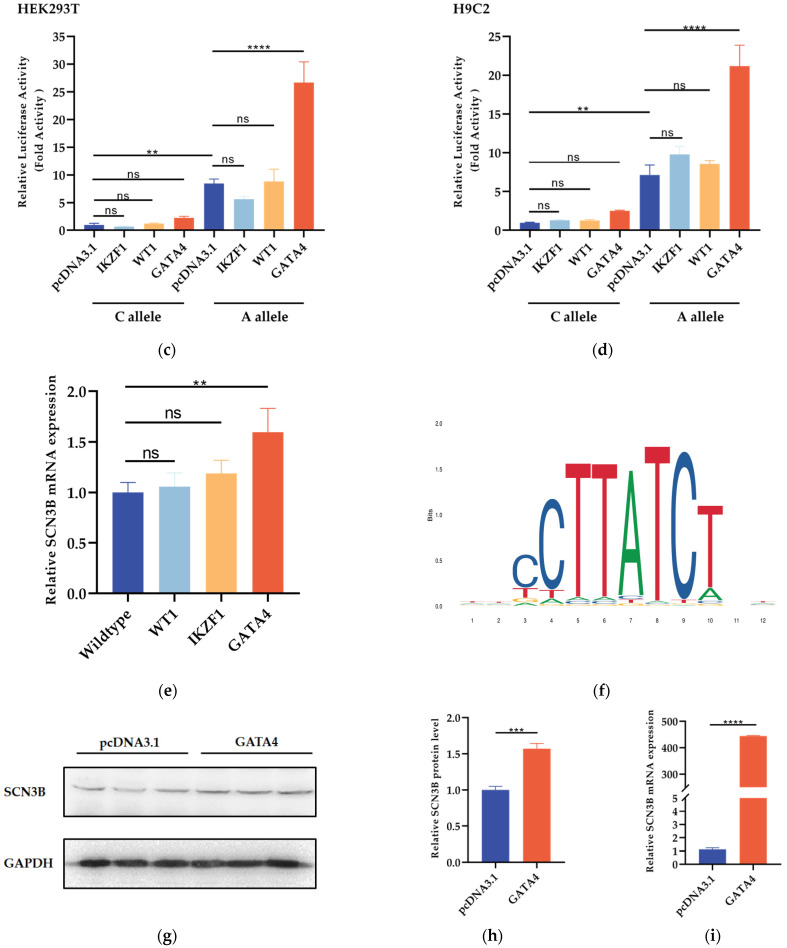
GATA4 positively regulates the expression of SCN3B. (**a**) Western Blot shows the overexpression degree of transcription factors IKZF1, WT1 and GATA4 in HEK293T cells; (**b**) above three transcription factors were overexpressed in H9C2 cells; (**c**) dual luciferase assay was performed when three filtered transcription factors including GATA4 overexpressed in HEK293T cells, respectively; (**d**) relative luciferase activities were measured when the above transcription factors were overexpressed in H9C2 cells; (**e**) *SCN3B* mRNA level among wild type, transcription factor WT1, IKZF1 and GATA4; (**f**) the putative binding site of GATA4 per human TFDB database, (**g**) Western blot exhibits the expression level of SCN3B when overexpressed GATA4; (**h**) qualification of *SCN3B* expression level from (**g**); (**i**) RT-PCR result shows *SCN3B* mRNA level when overexpressed transcription factor GATA4. (**c**–**e**) Analyzed by one-way ANOVA with Turkey’s post hoc test. (**h**,**i**) Analyzed by Student’s unpaired *t*-test. ** *p* < 0.01, *** *p* < 0.001, **** *p* < 0.0001, ns, not significan.

To investigate whether the discrepancy of transcriptional activity between the wild type and the variant c.-324 C>A (rs976125894) is affected by the transcription factors that bind on the surrounding of the c.-324 C>A, bioinformatics-based algorithms were applied to analyze and predict the potential transcription factors. The prediction results suggest that the alteration of C to A may change the motif of GATA4, WT1 and IKZF1. We overexpressed the above-mentioned three transcription factors in HEK293T cells and H9C2 cells, as shown in Figure 6a,b, and the relative luciferase activity was measured. We observed that among the three transcription factors, GATA4 manifested a relatively high luciferase activity in comparison with IKZF1 and WT1 in HEK293T cells and H9C2 cells (Figure 6c,d). To further validate the regulatory effects of IKZF1, WT1 and GATA4 on *SCN3B*, we overexpressed these three transcription factors in HEK293T cells. As shown in Figure 6e, the mRNA levels of *SCN3B* were upregulated by GATA4 (*p* < 0.05), while the other two transcription factors, IKZF1 and WT1, did not result in a significant difference. The putative binding site of GATA4 was predicted by JASPAR (Figure 6f). To determine whether the GATA4 would be capable of affecting the protein level of SCN3B, we overexpressed GATA4 in HEK293T cells. Interestingly, the expression level of both SCN3B protein and mRNA was increased by the overexpression of GATA4 (Figure 6g–i). These results showed that the GATA4 is a transcription factor that regulates the transcription of the *SCN3B* gene.

## 4. Discussion

In the present investigation, we carried out mutational screening for all exons and exon–intron boundaries of *SCN3B* and in a total of 355 AF patients, we found two rare variations, c.-324C>A (rs976125894) and c.-303C>T (rs1284768362), in the 5′UTR. We next examined whether the mutant alleles altered the regulatory features using bioinformatics and in vitro functional analyses. We discovered that variant c.-324C>A (rs976125894) in the *SCN3B* promoter region interacted with the transcription factor GATA4. The mutant A allele of the c.-324C>A (rs976125894) variant increased the promoter activity and may have resulted in abnormal overexpression of *SCN3B* when compared to the wild-type C allele. The most recent ACMG (American College of Medical Genetics) guideline classifies the c.-324C>A (rs976125894) variant as pathogenic when taken as a whole based on the extremely low frequency in the population database (PM2 Supporting), *de novo* via trio analysis (PS2), harmful prediction (PP3) and functional analysis (PS3). The disease-causing potential of the variant c.-303C>T (rs1284768362) is uncertain according to its ACMG categorization of variant of uncertain significance (VUS).

Four non-synonymous mutations (A130V, R6K, L10P and M161T) showed a decrease in the cardiac sodium current density and were discovered to be related to lone AF in previous studies [19,20]. However, the findings of the present investigation showed that a mutation in the 5′UTR of *SCN3B* is linked to lone AF and may be related to an increase in the expression level of the β3-subunit of the cardiac sodium ion channel (Na_V_1.5). Together, these results provide strong genetic support for *SCN3B*’s role as an AF susceptibility gene. The data also support the idea that rare mutations with major impacts play a significant role in the genetic underpinnings of AF.

More than 100 rare variants have been reported in patients with lone AF. All four previously described rare non-synonymous mutations in *SCN3B*—A130V, R6K, L10P and M161T—were electrophysiologically classified as loss-of-function mutations because they all decreased the cardiac sodium channel peak current density and impaired sodium current function. SCN3B A130V mutation might prevent the sodium channel Na_v_1.5 from trafficking to plasma membranes, R6K exerted a negative influence on steady-state inactivation, M161T caused a decrease in the peak current density and L10P affected both the steady-state inactivation and the sodium peak current density [15,19,20]. All these electrophysiological results support the hypothesis that the refractory period may be shortened by the decreased sodium current, thereby being attributed to AF susceptibility. In 2009, an *SCN3B* gene knockout mice model (*SCN3B*^−/−^) was applied to study atrial electrophysiology by Hakim et al. Compared with WT mice, the *SCN3B*^−/−^ mice exhibited abnormal ECG characteristics and irregular spontaneous atrial activity. Furthermore, *SCN3B*^−/−^ mice demonstrated a conduction abnormality and dysfunction of sinus node since the hearts of *SCN3B*^−/−^ mice show significantly longer sinus node recovery time than that of the WT hearts [21]. Valdivia and colleagues identified an *SCN3B* mutation V54G in a 20-year-old patient suffering from idiopathic ventricular fibrillation (IVF) and the mutation then caused a functional defect of *SCN3B* and reduced the sodium current, possibly via the mechanism of interfering with the normal localization of chaperone and cell membrane of *SCN3B*, thus, confirming that *SCN3B*, indeed, performs a significantly important role in maintaining the electric stability of the heart [22]. The above findings further accentuate the importance of *SCN3B* for the atrial function and conduction. Taken together, loss-of-function mutations in *SCN3B* may cause AF via shortening the cardiac refractory period or affecting the electric stability of the heart, indicating that *SCN3B* is AF causing.

Our findings may indicate, for the first time, that the variant increases the expression level of *SCN3B* or that gain of function can increase vulnerability to AF. The mutant A allele of the c.-324C>A variant (rs976125894) displayed an increased promoter activity and was positively controlled by GATA4. Together, the findings of the current study’s variant in the 5′UTR and the previously reported rare non-synonymous mutations showed that both gain-of-function and loss-of-function variants can provide a proarrhythmogenic substrate and increase the risk of AF. The same circumstances were seen in the *SCN5A* and *SCN1B* studies. In 2008, Ellinor and colleagues discovered that the *SCN5A* N1986K mutation led to a significant loss of sodium channel function and a prolongation of the atrial APD [23]. Although the most commonly postulated mechanism for the initiation of AF is a shortening of the atrial APD, an alternative mechanism may involve a prolongation of the atrial APD. Numerous studies linking *SCN5A* gain-of-function mutations with heightened vulnerability to AF lend credence to this theory. An *SCN5A* gain-of-function mutation (K1493R) that can increase cellular excitability and reduce the AP threshold has been found in AF patients, according to Li and colleagues [24]. Another gain-of-function mutation in *SCN5A*, M1851V, has been linked to ventricular arrhythmias and early-onset atrial fibrillation [25]. It has been found to reduce sodium channel current inactivation and accelerate recovery from inactivation. One gain-of-function *SCN1B* T189M mutation was found in patients with paroxysmal lone AF and when Na_V_1.5 and the T189M mutant Na_V_β1 subunit were co-expressed, electrophysiological analysis revealed that the expressed current density was significantly higher than that seen with the WT Na_V_β1 subunit [26]. Pilsicainide, a sodium-channel blocker, proved successful in treating AF in probands with gain-of-function variations in *SCN1B*. Additionally, it was discovered that the *PITX2c* M207V mutation caused familial AF and reporter gene assays showed that M207V increased *PITX2c*’s transactivation activity by 3.1-fold in comparison to its wild-type counterpart. This gain of function increased the mRNA levels of *KCNH2* (2.6-fold), *SCN1B* (1.9-fold), *GJA5* (3.1-fold), *GJA1* (2.1-fold) and *KCNQ1* in the homozygous form (1.8-fold) in the HL-1-immortalized mouse atrial cell line when the variant was co-expressed with wild-type *PITX2c* [23]. Taken together, gain-of-function mutations in sodium channels can cause AF, according to all our findings in the current study and other reports.

Genetic analysis, such as GWAS and candidate gene-based analysis, has identified a panel of SNPs associated with the risk of AF and most of them were found located within intergenic or intronic genomic regions [27]. Previous studies have demonstrated four mutations in the coding region of *SCN3B*; however, functional evidence about the mechanisms linking to non-coding variants with *SCN3B* or the incidence of AF is limited [19,20]. Recently, studies have suggested that SNPs predispose individuals to diseases via gradual and subtle functional differences rather than through significant biological alterations in functionality [28]. Therefore, for SNP rs976125894, a variant in the non-coding region we identified, we attempted to predict its potential functions through predicting the potential binding transcription factors, for instance, GATA4 and other functional studies, including promoter activity, enhancer activity and transcription factor binding activity.

Growing data suggest that a number of transcription factors, including GATA4, TBX5 and NKX2.5, play critical roles in cardiogenesis, recently conferring risk to AF or lone AF [29,30,31]. In our research, we found that the c.-324C>A mutation (rs976125894) in the 5′UTR of *SCN3B* caused an increase in transcriptional activity via interacting with transcription factor GATA4. GATA4 expression is seen throughout cardiac morphogenesis and is essential for normal heart development [32,33]. Patients with a wide range of congenital cardiovascular abnormalities have had the GATA4 gene mutation. GATA4 variants, such as G16C and p.H28D Y38D, P103A, M247T and A411V, have also been found in patients with lone AF, which is an interesting finding [34,35,36]. In addition, GWAS in large populations also discovered common variant rs35620480 in GATA4, conferring risk to common AF [37]. These findings suggest that a genetic predisposition to AF is conferred through impaired GATA4. Our research revealed a unique AF pathway by suggesting that one potential GATA4-linked mechanism of AF is the regulation of *SCN3B* expression levels.

The current study has some limitations. First, it is unknown if the variants in the current investigation impact the level of *SCN3B* expression in the myocardium because we were unable to obtain the patients’ human tissue. We examined man eQTL databases, including GTEX and eQTLGen, which can find the correlation between genetic variants and gene expression level in human tissues and obtained no results because of the extremely low frequency of these two variations and the lack of records for these two variants in these databases. Another limitation is that our functional work is preliminary and further investigation, such as functional electrophysiological analysis in induced pluripotent stem cells (iPS) or Crispr-Cas9 base-edited cells, will be required to establish the mechanism by which mutations in the 5′UTR of *SCN3B* cause atrial fibrillation.

In conclusion, we identified two rare variants in the 5′UTR and promoter region of *SCN3B* in patients with lone AF and discovered that one of them, the A allele of c.-324C>A (rs976125894), can improve transcriptional activity and may raise *SCN3B* expression levels. The A allele of c.-324C>A (rs976125894) has higher transcriptional activity when it interacts with GATA4, as we confirmed that the transcription factor GATA4 is a regulator of *SCN3B*. To the best of our knowledge, the current study is the first to demonstrate that the gain-of-function mutation of *SCN3B* can produce AF and the first to link a mutation occurring in the non-coding 5′UTR region of *SCN3B* to lone AF. The work also offers empirical proof that GATA4 is a critical regulator of *SCN3B* gene regulation. Our findings may serve as an encyclopedia for AF susceptibility variants and can also provide insight into the investigation of the functional mechanisms behind AF variants discovered by genetic methods.

## Figures and Tables

**Table 1:** Primers for Sanger DNA sequencing and HRM.

Primer		Sequence
rs976125894 (Sanger)	F	CCCCACTGGACCTCCCCAGT
	R	GATTCCAGTCGGAACGCAAC
rs976125894 (HRM)	F	CCTCCCCAGTTCGAGGGAGC
	R	CCACAGCCTGGCTGCTAGGC
rs1284768362 (Sanger)	F	CCCAGGGGGCGACTTTCTGA
	R	GAACGCAACCGATCCTGGGGA
rs1284768362 (HRM)	F	CCGATCAGCCGCTCCGCGCC
	R	AGCAGTGCGACTCCCTTCCGA

**Table 2:** Primers for qRT-PCR.

Target		Sequence
18S rRNA	F	CTCACTGAGGATGAGGTGG
	R	GTTCAAGAACCAGTCTGGGA
β-actin	F	GAAGATCAAGATCATTGCTCCTC
	R	ATCCACATCTGCTGGAAGG
SCN3B	F	TCTACTGGGTCAGTGTCTG
	R	CTTCATGCAGGAGATGCAG
GATA4	F	AGATGCGTCCCATCAAGAC
	R	CAGAGGCATTCAGGATGTG
WT1	F	CGTTTCTCACTGGTCTCAG
	R	ATCCTCCGAACCCAAAAGCC
IKZF1	F	AAGTTTCAGGGAAGGAAAGC
	R	CTCTTGGAGCTTTGCTGTC

**Table 3:** Characteristics of the GeneID lone AF population used for screening mutations in *SCN3B*.

Demographic and Clinical Feature	AF Population
Number of study subjects	355
Gender (number of females and %)	145 (40.85%)
Age (mean ± SD years)	44.05 ± 12.85

## Data Availability

Not applicable.
